# The Evolution and Expression Profiles of *EC1* Gene Family during Development in Cotton

**DOI:** 10.3390/genes12122001

**Published:** 2021-12-17

**Authors:** Xinyu Wang, Wei Chen, Jinbo Yao, Yan Li, Akwasi Yeboah, Shouhong Zhu, Yongshan Zhang

**Affiliations:** 1 Zhengzhou Research Base, State Key Laboratory of Cotton Biology, Zhengzhou University, Zhengzhou 450001, China; wxysst@163.com; 2 State Key Laboratory of Cotton Biology, Institute of Cotton Research, Chinese Academy of Agricultural Sciences, Anyang 455000, China; 15093906547@163.com (W.C.); yaojinbo@caas.cn (J.Y.); hai-19@163.com (Y.L.); akwasiyeboah97@gmail.com (A.Y.)

**Keywords:** cotton, egg cell 1 (*EC1*), evolution, gene family, double fertilization

## Abstract

Fertilization is essential to sexual reproduction of flowering plants. EC1 (EGG CELL 1) proteins have a conserved cysteine spacer characteristic and play a crucial role in double fertilization process in many plant species. However, to date, the role of *EC1* gene family in cotton is fully unknown. Hence, detailed bioinformatics analysis was explored to elucidate the biological mechanisms of *EC1* gene family in cotton. In this study, we identified 66 genes in 10 plant species in which a total of 39 *EC1* genes were detected from cotton genome. Phylogenetic analysis clustered the identified *EC1* genes into three families (I-III) and all of them contain Prolamin-like domains. A good collinearity was observed in the synteny analysis of the orthologs from cotton genomes. Whole-genome duplication was determined to be one of the major impetuses for the expansion of the *EC1* gene family during the process of evolution. qRT-PCR analysis showed that *EC1* genes were highly expressed in reproductive tissues under multiple stresses, signifying their potential role in enhancing stress tolerance or responses. Additionally, gene interaction networks showed that *EC1* genes may be involved in cell stress and response transcriptional regulator in the synergid cells and activate the expression of genes required for pollen tube guidance. Our results provide novel functional insights into the evolution and functional elucidation of *EC1* gene family in cotton.

## 1. Introduction

Fertilization is a complex phenomenon containing a series of orchestrating steps. In animals, before a spermatozoon can fertilize an oocyte, it undergoes physiological changes, and then band to develop a vigorous and intermittent flagellar movement [1,2]. Flagellated motile male gametes into the environment swim to non-motile eggs and finally, sperm cell fertilizes egg cell to gamete fusion. In plants, unlike animals, sperm cells in flowering plants are immotile. Furthermore, flowering plants perform double fertilization in which one sperm cell fuses with the egg cell and the resultant zygote develops into an embryo, the other fuses with the two polar nuclei in the central cell and grows into the triploid endosperm [3]. Double fertilization is crucial for the reproduction of flowering plants. It is highly dynamic and complex during the developmental process of double fertilization. During double fertilization, two immobile sperm cells are delivered to the chalazal edge of degenerated synergid cells between two female gametes, and the two sperm cells remain motionless together for a period of time and then fuse with egg cell and central cell [4]. The long quiescent period before the two sperm cells fuse with the female gamete indicates that there is an unclear cell–cell communication mechanism between the male gamete and the female gamete in flowering plants [5]. Flowering plants have evolved complicated mechanisms to assure and regulate every step during the pollen tube journey and subsequent double fertilization to maximize their reproductive success [6]. Recent studies have shown that some genes specifically expressed in gametophytes play an important role in the process of double fertilization. *DMP* (members of DOMAIN OF UNKNOWN FUNCTION 679 membrane protein) [7,8], *GEX2* (GAMETE EXPRESSED 2), and *HAP2*/*GCS1* (generative cell-specific protein) are expressed in the plasma membrane of sperm cells, and which are sperm-specific genes required for pollen tube guidance and fertilization [8,9,10]. Additionally, there is an egg-specific gene called *EC1*, it ensures the appropriate localization of the cell-fusion machinery in distinct sperm membrane domains and is involved in the attachment between male and female gametes in fertilized embryo sac and plays an important role in gamete activation [11]. In the process of gamete fusion, the down regulation of EC1 will reduce the speed of sperm activation or it may prolong the time frame needed to reach a certain EC1 threshold that is necessary to activate the sperm cells. The egg secretes EC1 protein when the sperm arrives to achieve rapid fusion with female gametes and preventing polytubey [12].

EC1 proteins, members of the ECA1 (early culture abundant 1) gametogenesis-related family proteins, belong to secreted small *CRPs* (cysteine-rich proteins) and *EC1* gene family and is the first known functional ECA1 gametogenesis-related family, which turned out to be essential for the double fertilization event [13]. EC1 proteins, characterized by a prolamin-like domain with six conserved cysteine residues may form three pairs of disulfide bonds and distinguishing marks of egg-cell-expressed EC1 proteins are, two short amino acid sequence motifs present in all EC1-like proteins [13]. Genes encoding EC1 proteins appear to be restricted to flowering plants and all five homologous *EC1* genes (*AtEC1.1*-*AtEC1.5*) in *Arabidopsis* show an egg-cell-specific expression pattern [13,14]. EC1 proteins are probably the most frequent *CRPs* secreted by the flowering plant egg cell and they are essential for gamete fusion in double fertilization [15]. In *Arabidopsis*, identified SUF4 (SUPPRESSOR OF FRIGIDA4) [16], a C2H2 transcription factor, acts as a regulator of the *EC1* gene expression. SUF4 binds to *EC1* promoters to regulate the expression levels of all five *EC1* genes. Furthermore, a down-regulation of *SUF4* was accompanied the down-regulation of *EC1* gene expression, impair rapid sperm fusion without abolishing it [17]. MOM1 (MORPHEUS MOLECULE1), CHD3-like chromatin remodeling protein, which has nucleosome-remodeling and histone deacetylation activities [16]. In *Arabidopsis mom1-3* ovules, both *SUF4* and *EC1* genes were down-regulated suggesting that MOM1 protein participates with *SUF4* in regulating the *EC1* genes. ChIP (chromatin immunoprecipitation) experiments revealed that *EC1* gene showed a higher level of histone 3-lysine-9 acetylation in *mom1-3* indicating that histone modifications also participate in *EC1* regulation [16]. In conclusion, EC1 protein plays an important role in the process of double fertilization in plants.

Cotton, cross-pollinated crop, is one of the most important textile fibers in the world. The release of different cotton whole-genome sequence data has provided an opportunity for genome-wide study of cotton-specific gene families [18,19,20,21] which offers an information for mining-related functional genes. Therefore, cotton is an ideal model for whole genome duplication (WGD) and evolution research. Although the function of EC1 protein in *Arabidopsis* has been studied, the comprehensive information and functional characterization of cotton *EC1* gene family are still unclear, as well as how the said gene regulates pollination and fertilization process of cotton is unknown. Owing to the above reasons, this present study was conducted to investigate EC1 proteins in cotton using bioinformatics methods and to unravel the gene structure features, chromosomal locations, phylogenetic relationships, synteny and expression patterns to highlight the potential functional diversity. Our results will provide useful theoretical support for the functional characterization of the *EC1* genes that are involved in gamete fusion in double fertilization process in cotton.

## 2. Materials and Methods

### 2.1. Identification and Characterization of EC1 Gene Family Members

The amino acid sequences of 5 AtEC1 protein as queries in searches against the database of other plant species (*Gossypium arboreum* (ICR, version 1.0), *Gossypium hirsutum* (ZJU, version 2.0), *Gossypium raimondii* (JGI, version 2.0), *Gossypium barbadense* (ZJU, version 2.0), *Sorghum bicolor* (version 3.1.1), *Oryza sativa* (version 7.0), *Populus trichocarpa* (version 4.1), *Theobroma cacao*, (version 2.1), and *Vitis vinifera* (version 2.1)), respectively. The databases with the most up-to-date genome annotation data were downloaded from Phytozome (version 13) (https://phytozome.jgi.doe.gov/pz/portal.html, accessed on 22 November 2020) for all species except for *Arabidopsis* and cotton. The genome database of *Arabidopsis* and cotton were downloaded from TAIR (https://www.arabidopsis.org/, accessed on 20 October 2020) and CottonFGD (https://cottonfgd.org/, accessed on 26 November 2020), respectively and BLASTP (Protein-protein Basic Local Alignment Search Tool) was performed. The conserved domain database (cdd) (https://www.ncbi.nlm.nih.gov/cdd/, accessed on 14 December 2020) and SMART tool databases (http://smart.embl-heidelberg.de/, accessed on 24 December 2020) were used to detect the domains of all candidate gene protein sequences. *EC1* genes of other plants were also obtained according to this method. The names of all the genes were showed in Table S1. The physical and chemical properties such as the isoelectric point molecular weight of the identified *EC1* were analyzed with ExPASy ProtParam tool (http://us.expasy.org/tools/protparam.html, accessed on 10 March 2021). Subcellular localization prediction was used using Softberry online website (http://www.softberry.com, accessed on 20 March 2021) [22].

### 2.2. Phylogenetic Analysis of EC1 Genes

To analyze evolutionary relationships, phylogenetic trees were constructed with MEGA7.0 software using the neighbor-joining method [23], with default parameters and bootstrap test was executed by 1000 replications.

### 2.3. Chromosomal Locations and Gene Collinearity Analysis

The diagram of the chromosomal locations was plotted using TBtools software [24]. Genome assembly sequence, gene annotation files of all four species were downloaded from CottonFGD (http://www.cottonfgd.org/, accessed on 10 May 2021). Systematic names of *EC1* genes were assigned based on their chromosome distribution. Whole proteins of the four cotton species were compared against each other using local blast software with e value less than e^−5^. The blast outputs of all protein-coding genes were imported into MCScanX [25]. The collinear relationships of duplicated *EC1* genes in four cotton species *G. arboreum*, *G. barbadense G. hirsutum*, and *G. raimondii* were obtained by MCScanX. The syntenic relationships of *EC1* genes were demonstrated in a circos map using CIRCOS software (http://circos.ca, accessed on 13 July 2021).

### 2.4. Gene Structure and Multiple Sequence Alignment Analysis

The exon-intron structure of *EC1* genes was determined by comparing predicted coding sequences with their corresponding full-length sequences using the online program Gene Structure Display Server (GSDS: http://gsds.cbi.pku.edu.cn, accessed on 25 June 2021) [26]; it was based on the alignment of the CDS with their corresponding genomic DNA sequences. Conserved protein motifs of the EC1 proteins were predicted by the MEME program (http://meme-suite.org/tools/meme, accessed on 15 March 2021) [27]. The maximum motif search value was set at 10 and the rest of the parameters was set to default. The SMART tool databases (http://smart.embl-heidelberg.de/, accessed on 22 March 2021) were used to detect the domains of all EC1 protein sequences. Multiple sequence alignment of deduced EC1 proteins was performed using Geneious prime with default parameters. The three-dimensional protein structure was generated using SWISS-MODEL (https://swissmodel.expasy.org/, accessed on 22 September 2021) [28] and the template file obtained from AlphaFold Protein Structure Database (https://www.alphafold.ebi.ac.uk/, accessed on 22 September 2021) [29].

### 2.5. Promoter Regions Analysis of EC1 Genes

For the identification of cis-regulatory elements in *EC1* genes, we retrieved an upstream sequence from CottonFGD (http://www.cottonfgd.org/, accessed on 10 April 2021) of 2 kb of our genes from the translation start site. Subsequently, the sequences obtained were searched in PlantCare database (http://bioinformatics.psb.ugent.be/webtools/plantcare/html/, accessed on 12 April 2021) [30] and eventually various cis-acting regulatory elements for our sequences were predicted.

### 2.6. Analysis Gene Expression

RNA-seq data of different tissues expression and stress treatment (under cold, heat, salt, and PEG) of *GhEC1* genes were obtained from the Cotton Omics Database (COD) (http://cotton.zju.edu.cn/, accessed on 25 April 2021) [20,21]. The RNA-seq data were assessed by quantitative RT-PCR (qRT-PCR). The total RNA was isolated by using RN38-EASYspin-Plus Plant RNA Kit (Aidlab Co., LTD, Beijing, China). The total RNA was reverse-transcribed using PrimeScript™ RT reagent Kit with gDNA Eraser (Takara Biomedical Technology Co., LTD, Beijing, China) according to the manufacturer’s instructions. All the primer sequences are shown in Table S2. qRT-PCR assays were performed on the Bio-Rad 7500 fast fluorescence quantitative PCR platform with SYBR^®^ Premix Ex Taq^TM^ (Tli RNaseH Plus) (Takara Biomedical Technology Co., LTD, Beijing, China) in accordance with the manufacturer’s protocol. *GhActin* gene was selected as an internal reference gene. The experiments were independently repeated three times, and 2^-Δct^ method was used to calculate relative expression levels of *GhEC1* genes.

### 2.7. Interaction Network of GrEC1 Proteins

STRING software (https://string-db.org/, accessed on 10 May 2021) was used to analyze the interaction of GrEC1 proteins with a confidence parameter set at 0.15 threshold [31]. We performed STRING network analysis using protein families and multiples search. The protein sequences of GrEC1 were used for interactive network search. The string interaction predicts protein–protein interactions based on co-citation in the literature, co-regulation of gene expression, gene co-occurrence or gene neighborhood.

## 3. Results

### 3.1. Identification of EC1 Genes

BLASTP analysis was performed to search for *EC1* genes from two monocotyledons and eight dicotyledons. Based on the analysis, three *EC1* genes in *Oryza sativa* and two in *Sorghum bicolor* (Monocots) while six in *Populus trichocarpa*, four in *Vitis vinifera*, seven in *Theobroma cacao*, five in *Arabidopsis*, six in *G. arboreum*, 13 in *G. barbadense*, 14 in *G. hirsutum,* and six in *G. raimondii* (Dicots) were identified. To ensure the precision of initial results, we used the cdd (cdd/wrpsb.cgi) and SMART tool databases (http://smart.embl-heidelberg.de/, accessed on 24 December 2020) to detect the domains of all candidate gene protein sequences for further confirmation. For four cotton species, a total of 39 *EC1* genes were identified and were named based on the order of genes located in chromosomes. We found an abnormal sequence of four genes (*GH_A05G1455*, *GH_D05G1474*, *GB_A05G1471*, *GB_D05G1480*) in the database based on alignment with other cotton sequences and combining with data from other cotton databases (https://www.cottongen.org/) [30,32], we determined that the gene and protein sequences in the cotton databases (ZJU, version 2.0) were incorrectly annotated. Corrections were made for the sequences of genes in this study (Table S1).

Subsequently, the biophysical properties of the *EC1* genes in four cottons including genomic position, transcript features, and protein statistics were determined (Table S1). All of *EC1* genes identified above were named based on their chromosomal location. Ga, Gb, Gh, and Gr were used as prefixes before the names of *EC1* genes from *G. arboreum*, *G. barbadense*, *G. hirsutum,* and *G. raimondii*, respectively. The identified *EC1* genes in cotton-encoded proteins were mainly amino acids which varied from 123 to 213, with a wide variation of CDS (372 to 642 bp), PI (4.17 to 8.91), and MWs (13.10 to 23.18 (kDa)) (Table S1).

### 3.2. Phylogenetic Analysis of EC1 Gene Family in Cotton

**Figure 1:**
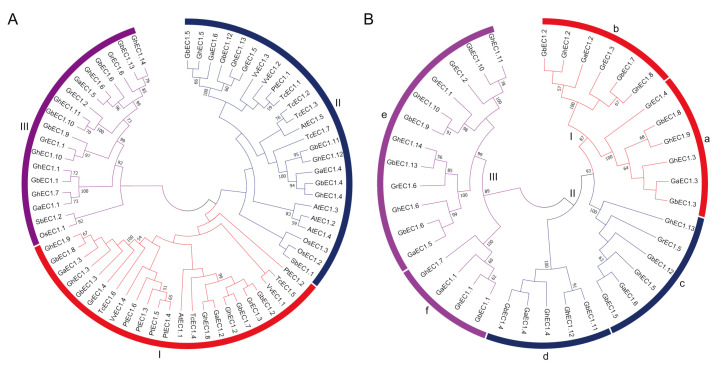
Phylogenetic tree of the EC1 family proteins. The unrooted tree was generated using the MEGA7.0 program with the neighbor-joining (NJ) method. Bootstrap values from 1000 replicates are indicated at each branch. (**A**) The phylogenetic trees were constructed based on the full-length protein sequences of the EC1 proteins from *Oryza sativa*, *Vitis vinifera, Sorghum bicolor*, *Populus trichocarpa*, *Theobroma cacao*, *Arabidopsis*, *G. arboreum*, *G. barbadense*, *G. hirsutum*, and *G.* *raimondii*. (**B**) The phylogenetic trees were constructed based on the full-length protein sequences of the EC1 proteins from *G.* *arboretum*, *G. barbadense*, *G. hirsutum* and *G. raimondii*.

The phylogenetic analysis of EC1 proteins were established based on protein sequences of *Oryza sativa*, *Vitis vinifera Sorghum bicolor* while *Populus trichocarpa*, *Theobroma cacao*, *Arabidopsis*, *G. arboreum*, *G. barbadense*, *G. hirsutum*, and *G. raimondii*. The EC1 proteins were clustered into three distinct groups with Group II containing the highest members (25), followed by Group I (23), and Group III (18) with the lowest members (Figure 1A). Interestingly, four members of the EC1 proteins in *Arabidopsis* were clustered in Group II. The monocots (*Oryza sativa* and *Sorghum bicolor*) showed a completely different distribution pattern and the species were grouped into Groups II and III. To investigate the evolutionary relationship of EC1 proteins among different cotton species, phylogenetic tree was constructed (Figure 1B). To further study the evolutionary relationship and to predict the gene function, the *EC1* genes in cotton were divided into six subgroups, named a-e, respectively (Figure 1B). The *EC1* gene was divided into three major groups comprising six subgroups (Figure 1) with Group III-e as the largest subgroup (12 EC1 members) and Group III-f as the smallest subgroups with only four members. On the basis of these results, we speculated that this result is due to whole genome duplication in the process of evolution of cotton.

### 3.3. Chromosomal Locations and Gene Collinearity Analysis

**Figure 2:**
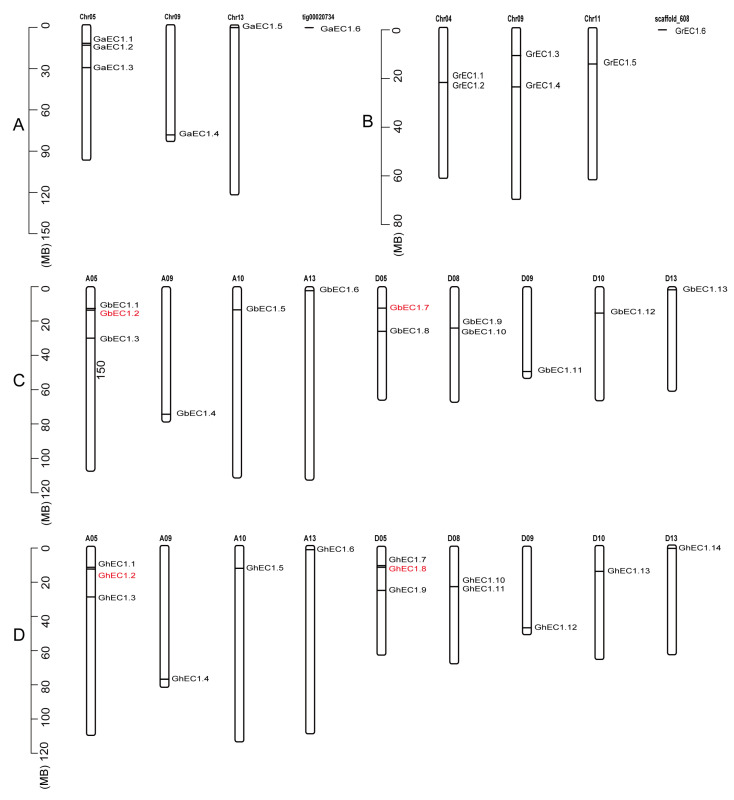
Chromosomal localization of *EC1* genes on *G. arboretum* (**A**), *G. raimondii* (**B**), *G. barbadense* (**C**) and *G. hirsutum*. (**D**). The scale represents megabases (Mb). The chromosome numbers are indicated above each vertical bar. Gene names after correction of annotation errors are indicated in red.

To determine the chromosomal distribution of *EC1* genes and better understand the mechanism of genomic distribution, chromosomal localization maps were constructed from *G.* *arboretum*, *G. barbadense*, *G. hirsutum*, and *G. raimondii*. Among six identified *EC1* genes of *G.* *arboretum*, five members were distributed on three chromosomes, while only one gene was found at scaffold region (Figure 2A). Chromosome chr05 contains three of *EC1* genes in *G.* *arboretum* while chr13 and chr09 contains only one gene. Five out of six identified *EC1* genes from *G. raimondii* were assigned to three chromosomes with only one *EC1* gene on scaffold (Figure 2B). Total of 13 identified *EC1* genes from *G. barbadense* were assigned to nine chromosomes. Six and seven genes were localized to the four chromosomes from At subgenome and five chromosomes from Dt subgenome, respectively (Figure 2C). In the identified *EC1* genes from *G. barbadense*, *GbEC1* genes were evenly distributed among all the chromosomes, and the distribution of *EC1* genes on a single chromosome was one or two genes. Similarly, 14 *EC1* genes from *G. hirsutum* were mapped on nine chromosomes. At subgenome harbors six genes and Dt Subgenome contains eight *EC1* genes (Figure 2D) and the distribution pattern of these genes on the chromosomes was the same as that of *G. barbadense*. The chromosomal positions of *GbEC1.2* (A05: 13711970-13718094), *GbEC1.7* (D05: 12487956-12494073), *GhEC1.2* (A05: 13322117-13328241) and *GhEC1.8* (D05: 12247842-12253949) genes were incorrectly annotated, so we corrected them. The correct positions of *GbEC1.2*, *GbEC1.7*, *GhEC1.2* and *GhEC1.8* are as follows: A05:13717681-13718094, D05:12493660-12494073, A05:13327828-13328241, and D05:12253536-12253949, respectively (Figure 2).

**Figure 3:**
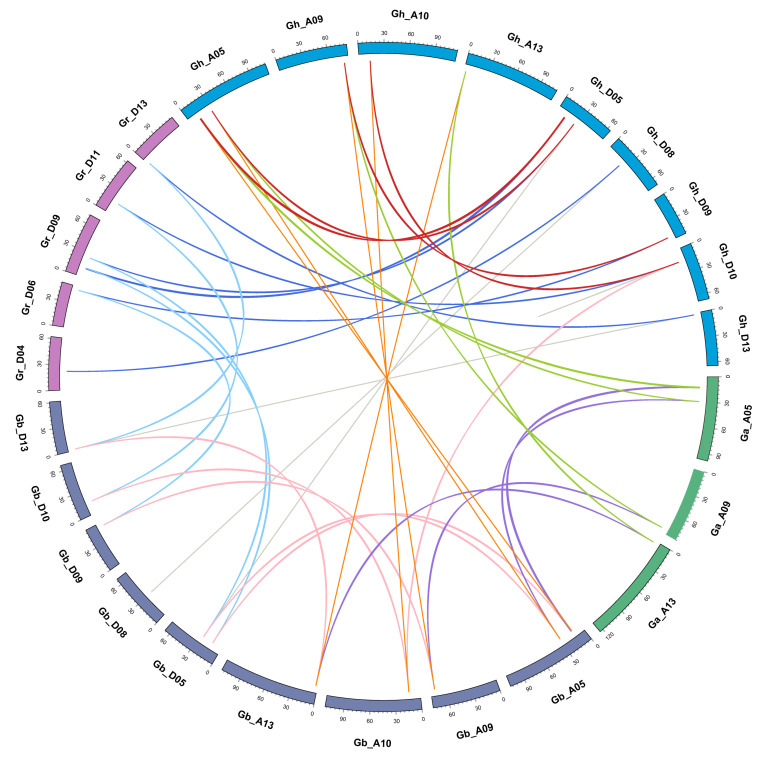
The collinearity relationships of *EC1* genes in cotton. The scale on the circle is in Megabases. Each colored bar represents a chromosome as indicated. The green and purple lines represent the collinearity of the At subgenome to the A subgroup of *G. hirsutum* and *G. barbadense,* respectively; dark blue and light blue lines represent the collinearity of the Dt subgenome to the D subgroup of *G. hirsutum* and *G. barbadense*, respectively; red and pink lines represent the collinearity between subgroup A and D in *G. hirsutum*; and *G. barbadense,* respectively; and both gray and orange lines represent the collinearity of the D subgroup of *G. hirsutum* to the D subgroup of the *G. barbadense*.

**Figure 4:**
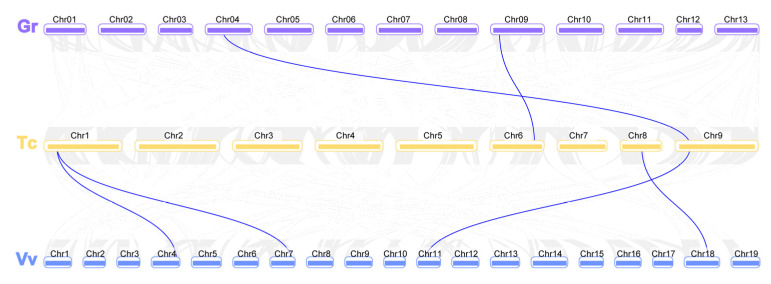
The synteny and collinearity analysis of *EC1* genes among grape, cacao, and cotton. The positions of *EC1* genes were depicted in the chromosome of *V. vinifera* (Vv, blue band), *T. cacao* (Tc, yellow band), and *G. raimondii* (Gr, purple band).

Gene family expansion occurs via the following three mechanisms: whole genome duplication, tandem duplication, and segmental duplication. Gene duplications, occurring during the course of evolution, have led to the diversity of gene function. Based on chromosome distance, coverage, and similarity, the evolutionary relationship between *EC1* gene families in diploid and tetraploid cotton was analyzed. To reveal the mechanism(s) behind the expansion of the *EC1* family in four cottons, all intragenomic and intergenomic duplication data files of four cotton species were filtered by MCScanX and analyzed. A total of 42 orthologous/paralogous gene pairs were identified of which most of them were whole genome duplication (WGD). In this study, five pairs of orthologous/paralogous gene pairs were identified among At and Dt subgenomes of both allotetraploid cotton species (*G. hirsutum*, *G.* *barbadense*) respectively (Figure 3, Table S4). Among the five pairs of duplicated *GhEC1* genes, all were observed between different chromosomes and all pairs were identified between the At and Dt subgenomes. The analysis showed that several *EC1* gene loci were highly conserved between the At and Dt sub-genomes of both allotetraploid cotton species. Collinear relationship among the orthologous *EC1* genes from grape, cacao, and cotton were surveyed to investigate the putative clues of evolutionary events. As observed, there were *EC1* syntenic relationships among the collinearity of grapes, cocoa, and cotton (Figure 4). The analysis showed that several *EC1* genes were highly conserved. In two allotetraploid cotton species, the number of *EC1* genes was much higher than that of any other plant species, including the two diploid cotton species with their progenitors *G. arboreum* and *G. raimondii*. From these results we presumed that WGD is the major gene duplication modes of *EC1* genes family.

### 3.4. Exon-Intron Structure, Motif and Sequence Analysis

**Figure 5:**
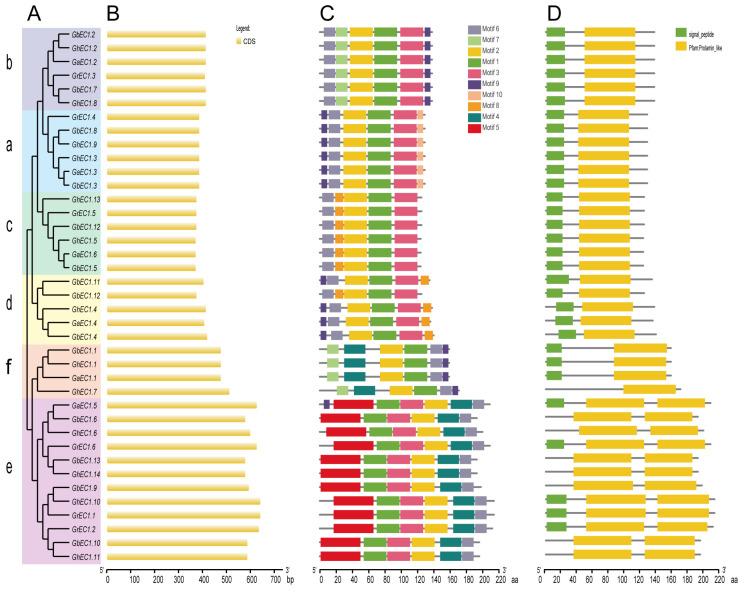
Phylogenetic relationship, exon-intron organizations, and motif analysis of EC1 proteins. (**A**) Phylogenetics tree of EC1 proteins from four cottons. Phylogenetic tree was created in MEGA7.0 software with the neighbor-joining (NJ) method. Bootstrap values from 1000 replicates are indicated at each branch. The phylogenetic trees were constructed based on the full-length protein sequences of the EC1 proteins from *G. arboreum*, *G. barbadense*, *G. hirsutum*, and *G. raimondii*. (**B**) Gene structure (exon-intron) analysis of *EC1* genes. (**C**) Motif prediction of EC1 proteins. Ten motifs were identified by MEME online tool. The length and position of the motifs could be estimated according to the scale bar. (**D**) Domain prediction of EC1 proteins.

In plants, most genes are interrupted genes that have one or more exons and several introns. The arrangement of introns and exons in gene families can provide valuable information regarding the evolutions and functions of gene families. In this study, we analyzed the exon and intron arrangement among different members of *EC1* gene family to further explore its phylogenetic relationship. To better understand the diversity and similarity of gene structure and motif among EC1 proteins, we constructed a separate phylogenetic tree and visualization using GSDS with Figure 5B showing the exons. All gene structures of *EC1* family had only one exon without intron and with a conserved domain.

In the phylogenetic tree, most tightly clustered *EC1* genes showed a similar length of exon (Figure 5A). We performed MEME program [27] for the discovery of motifs in all classes and extracted 10 motifs at default parameters of MEME. As expected, the *EC1* members were gathered close to each other in the same clade demonstrating similar patterns of conserved motif distribution. Most members of the *EC1* family had five to six conserved motifs. All members of the EC1 proteins contained motif 2 and motif 7 (Figure 5C). Some motifs were present in some specific classes. For example, Motif4 was conserved in Group III-e and Group III-f EC1 proteins. We speculated that the unique motif of different clades may represent the conserved and specific function of the *EC1* gene family. In addition, the motifs and their arrangement in the EC1 proteins were alike among proteins within the same class, signifying a common origin and/or close relationship. Most family members had the signal peptide at the N-terminal suggesting that EC1 proteins are related to cell secretion (Figure 5D). There were several genes without signaling peptides which may be due to the gene changes in the process of evolution.

**Figure 6:**
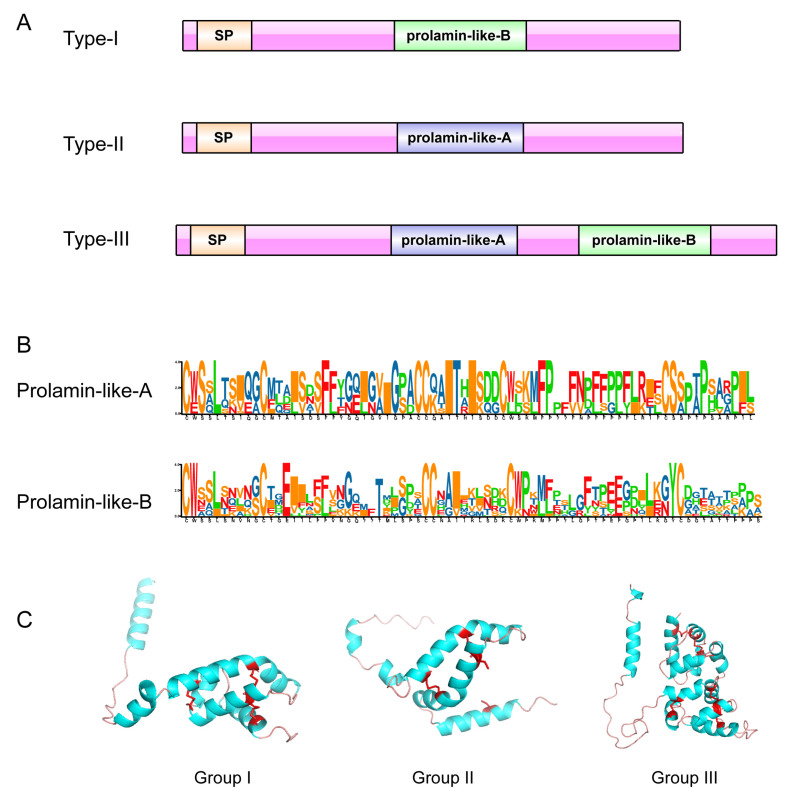
Multiple sequence alignments and the conserved domains of EC1 proteins from four cotton (**A**) The domains were highlighted by green and purple boxes. (**B**) Sequence logo was generated by TBtools software. (**C**) Three-dimensional structure of three EC1 proteins (GroupI (*GrEC1.3*), GroupII (*GrEC1.5*) and GroupIII (*GrEC1.1*)). Conserved cysteine sites are highlighted in red.

Sequence analysis of the deduced proteins showed a conserved domain prolamin-like, which is a characteristic of the major facilitator superfamily. The relatively conserved tryptophan residues were found after the first and fifth cysteines residue (Figure 6B and Figure S1). The sequence’s characteristic is the same as that reported for *Arabidopsis EC1* genes [13]. The most conserved residues were located in the two larger cysteine-loops that is the second and third cysteine with the fourth and the fifth cysteine in between. The *EC1* gene family belongs to a subfamily *CRP* gene family. Intact *CRPs* are highly cross-linked by multiple disulfide bridges which confer their high stability. Therefore, *CRPs* can be classified according to their specific “disulfide signature” [33]. Cysteine residues are often conserved in evolutionarily related proteins that have similar structures [34]. Most members of the *CRP* gene family have a scaffold of eight conserved cysteines, but the EC1 proteins have six cysteines, which would cause protein structure variation. Protein structure and its function are interrelated, and any change in protein structure may affect its function. Despite differences in sequence, members of this superfamily possess the characteristic conserved skeleton of six cysteine residues as demonstrated in all sequences. We further analyzed the conservation of the homologous domain of these proteins and found three types of EC1 proteins (Type-I/-II-/III) (Figure 6A). Most EC1 proteins had only one Prolamin-like domain and a few of them with two Prolamin-like domains. Sequence alignments of the domains revealed two differences between Prolamin-like-A and Prolamin-like-B (Figure 6B). Interestingly, type-III *EC1* genes forming Group III-e were found only in cotton which may be attributed to the fusion or recombination events during the evolution of these genes, hence, indicating the importance of these genes. Type-II genes formed Group III-f and type-I formed Group I and Group II. For a better understanding of the structure, one gene was randomly selected from each group (Group I (*GrEC1.3*), Group II (*GrEC1.5*), and Group III (*GrEC1.1*) shown in Figure 6C) to predict its three-dimensional structure.

### 3.5. Cis-Elements Analysis of GhEC1 Genes Promotors

**Figure 7:**
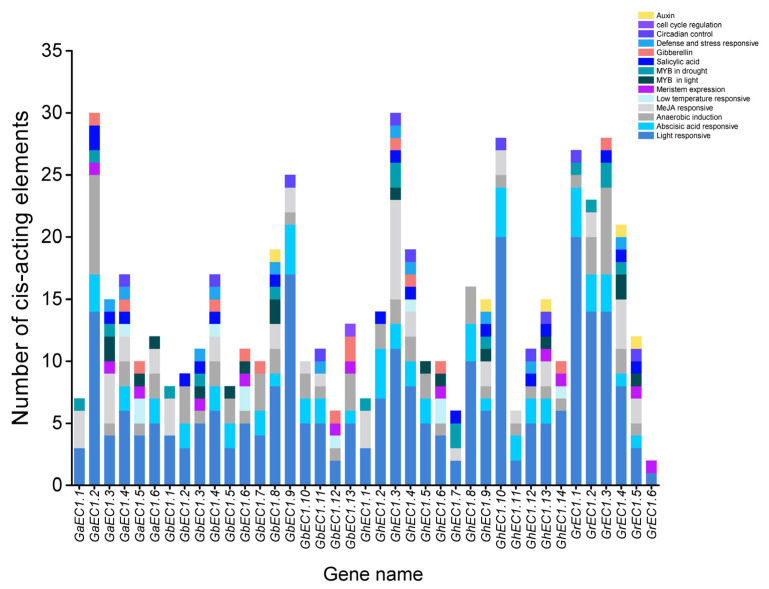
Cis-elements of the *EC1* genes. Vertical axis represents the number of cis-elements, and the horizontal axis shows the genes name.

Gene expression is controlled by their interaction of transcription factors and target cis-elements DNA elements in promoters. The cis-elements in the promoter regions of genes provide an important insight into the stress response of plants to define the tissue-specific expression behavior or stress receptive under different environments. Therefore, to understand the mechanisms behind abiotic stress tolerance, the promoter sequence of *GhEC1* genes was analyzed for identification, extraction, and scanning of stress-related and hormone-responsive cis-elements using PlantCARE database. Our analysis detected a large number of cis-elements in the promoter of *GhEC1* genes (Figure 7). The cis-elements identified in our study can be classified into four main types. First, five hormone-responsive regulatory elements in the increasing order namely abscisic acid responsiveness (ABRE), methyl jasmonate (MeJA), gibberellin (GA), salicylic acid (SA), and auxin (IAA) responses were identified in the *GhEC1* promoters. The second types of regulatory element found in the *GhEC1* promoters were stress-related cis-elements including cis-element involved in low-temperature responsiveness (LTR), MYB binding site involved in light responsiveness and drought-inducibility (MBS), cis-element involved in defense and stress responsiveness (TC-rich repeats), and anaerobic induction (ARE). Among them, ARE cis-elements were most frequently found, followed by MBS, LTR, and TC-rich repeats. The third type of regulatory element found in the *EC1* promoters were growth and development cis-elements, involved in cell cycle regulation (MSA-like), circadian control (circadian), and meristem expression (CAT-box). CAT-box covered the largest portion of the third category of cis-elements, followed by circadian, and MSA-like. The fourth group was the light-responsive regulatory elements which comprised of different elements predicted in the *GhEC1* genes. We speculated that some *EC1* genes might have the potential to improve abiotic stress responses.

### 3.6. Expression Profile of Cotton EC1

**Figure 8:**
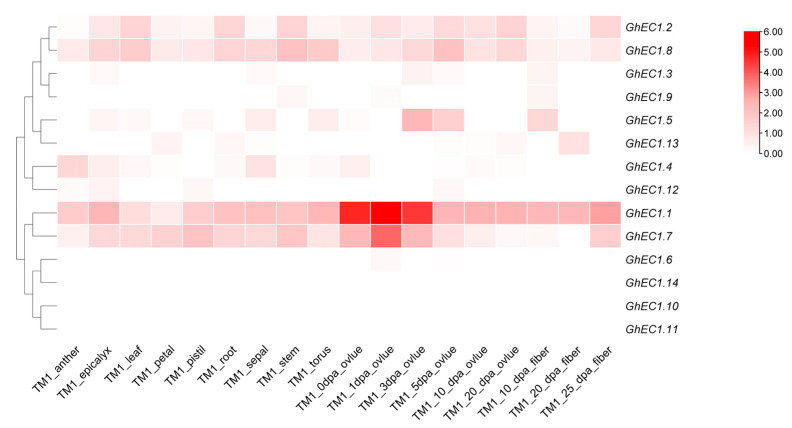
The expression profiles of *GhEC1* genes. The heatmap was generated based on RNA-seq data from the website (http://cotton.zju.edu.cn/, accessed on 25 April 2021), the color scale was shown at the right of the figure.

To better understand the function of *GhEC1* genes, publicly available transcriptome datasets were analyzed. The heat map shows that the same subgroup showed similar expression patterns (Figure 8; Table S5). Most of the *GhEC1* genes were expressed in ovules, while one gene (*GhEC1.14*) was not expressed in almost all tissues and organs. Group I and II included *GhEC1.3*, *GhEC1.4*, *GhEC1.5*, *GhEC1.9*, *GhEC1.12*, and *GhEC1.13* which were highly expressed in ovules, fibers, and slightly expressed in some other tissues. The expression of *GhEC1.2* and *GhEC1.8* was different from that of the group, and exhibit constitutive expression patterns in different tissues. In Group III, two genes (*GhEC1.1* and *GhEC1.7*) of Group III-f were expressed in almost all tissues and in ovules, suggesting the role of these genes in regulating reproductive development. Among the four genes (*GhEC1.6*, *GhEC1.10*, *GhEC1.11*, and *GhEC1.14*) in Group III-e, *GHEC1.14* was almost not expressed in tissues and organs and the other three genes were only slightly expressed in the ovules.

**Figure 9:**
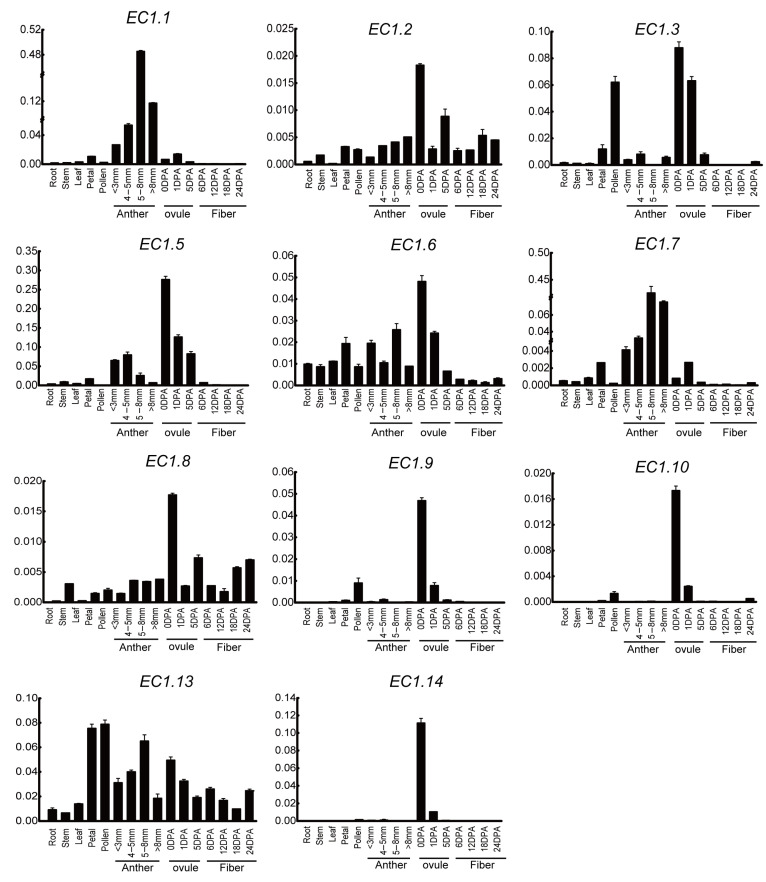
The expression patterns of *EC1* genes in *G.hirsutum*. qRT-PCR was conducted to analyze the relative expression of 11 *EC1* genes in root, stem, leaf, petal, pollen, anther, ovule, and fiber. *GhActin* gene was selected as an internal reference gene. qRT-PCR experiments were performed with three independent replicates, and the error bars in this figure represent the SDs from three independent experiments.

To verify the accuracy of RNA-seq data, qRT-PCR of 11 randomly selected *EC1* genes in *G. hirsutum* was performed to determine the expression pattern in root, stem, leaf, petal, pollen, anther (flowers bud size <3 mm, 4–5 mm, 5–8 mm and >8 mm, the classification method of anthers at different developmental stages was described by Koltunow [35] and Scott [36]), ovule and fiber (Figure 9). The qRT-PCR results showed that almost all genes were expressed in ovules and followed a similar pattern with that detected by the RNA-seq data, except *EC1.2* and *EC1.8*, which may be due to the incorrect annotation of gene and protein sequences in the ZJU, version 2.0 databases. Similarly, most *EC1* genes were highly expressed in the anthers and reproductive tissues, indicating the functioning of the gene in promoting the ovule and anthers at different developmental stages. In addition, *EC1.1* and *EC1.7* genes were highly expressed in the anthers, especially in 5–8 mm anthers. We infer the function of EC1 through a combination of their expression and the function of their orthologs in model plants, which provided an efficient approach to screen potential candidates for further functional studies.

In addition, the expression changes of each gene were different under different abiotic stresses. To further investigate the potential functions *EC1* genes in response to abiotic stresses in cotton, we analyzed the *G. hirsutum* transcriptomic data to detect the *GhEC1* genes involved in heat, salt, polyethylene glycol (PEG), and cold stress (Figure S2; Table S6). *GhEC1.2*, *GhEC1.8*, *GhEC1.1*, and *GhEC1.7* showed expression in response to multiple abiotic stresses. When the cotton plants were exposed to three abiotic stresses, nearly half of the members were less or almost imperceptibly expressed especially those in Group I and II. Genes of Group III-f were barely expressed at all time points. Homologous genes such as *GhEC1.13* and *GhEC1.5*, were continuously expressed upward after cold stress and drought stress. *GhEC1.12* and *GhEC1.4* genes were continuously expressed upward after heat stress. All four genes belong to Group II, suggesting that members of the same group showed not only functional redundancy but also functional differentiation.

### 3.7. Interaction Network of GrEC1 Proteins

**Figure 10:**
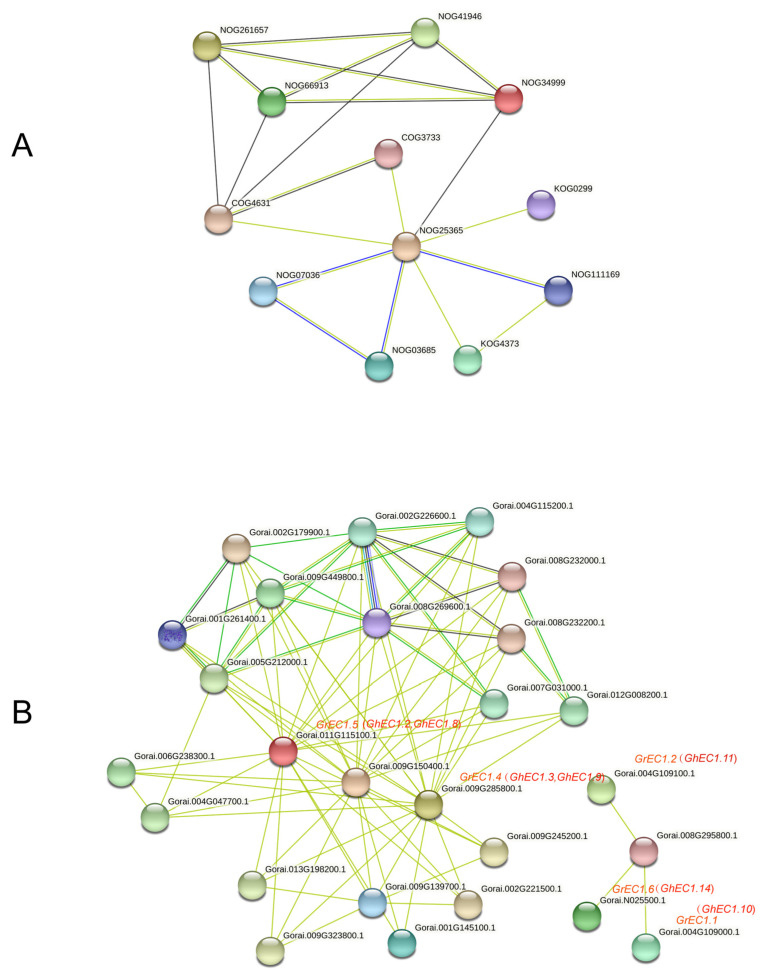
Interaction network of EC1 proteins (**A**) Interaction network of EC1 proteins families. (**B**) Interaction network of GrEC1 proteins with other proteins. The light green lines represent the protein–protein interaction based on textmining; dark green lines represent the protein–protein interaction based on gene neighborhood; black lines represent the protein–protein interaction based on co-expression; blue lines represent the protein–protein interaction based on gene co-occurrence; purple line represent the protein–protein interaction based on protein homology.

Protein interaction network provides information for understanding protein function and is the basis for understanding protein function [37,38]. To understand the functional relationships among these EC1 proteins, we performed an interaction network analysis by STRING data (https://string-db.org/, accessed on 10 May 2021) using protein families (Figure 10A) and multiple sequences (Figure 10B) search. By the method of protein families search, the proteins from *G. raimondii*, involved in regulation of double fertilization forming a zygote and endosperm pathway (NOG25365) and Prolamin-like pathway (NOG34999) were identified. These proteins belong to the small secretory protein family. In addition, proteins of disruption of cells of other organisms (NOG41946) and self pollen (NOG66913) also belong to the small secretory protein family. These proteins are extracellular signaling molecules that may act during embryo sac development or fertilization [39]. These results are consistent with our analysis of the EC1 proteins. These results predicted that EC1 proteins in cotton may play an important role in certain cell interactions during double fertilization. Proteins interaction network query was performed by using *EC1* member sequence of *G. raimondii*. As is shown in Figure 10B, GhEC1.3, GhEC1.9, GhEC1.2, and GhEC1.8 interacted with Gorai.001G145100.1. These proteins have not been studied in cotton, but their homologous genes were identified candidate genes involved in gametic and/or early zygotic development in rice [40]. As is shown in Figure 10B, *GhEC1.10*, *GhEC1.11*, and *GhEC1.14*, interacted with *Gorai.008G295800.1* and did not form an interaction network with other proteins. Interestingly, *GhEC1.10*, *GhEC1.11*, and *GhEC1.14* belong to Group III-e and have a unique motif 5 compared with members of other groups. This means that these three members have the potential to have some special functions. *GhEC1.2 GhEC1.8*, *GhEC1.3*, and *GrEC1.9* form an interaction network. In addition, homologues of these genes were identified as candidate genes involved in double fertilization in *Arabidopsis* [39]. The results suggest that it may act as signals between the male gamete and the female gamete during fertilization.

## 4. Discussion

Recently, small cysteine-rich proteins have remarkably attracted the attention of researchers. It has been shown that small cysteine-rich proteins also have a role in abiotic stress, exhibit potent antifungal activity in vitro, and are involved in short-range signaling required for pollen tube attraction by the female gametophyte [41,42,43]. Previous studies on the EC1 family have focused mainly on *Arabidopsis*. In this study, we first reported on the *EC1* gene family in cotton in which 39 *EC1* members were identified which is higher than that reported in others. The *EC1* gene family in cotton significantly expanded with cotton tetraploid event. The results indicated that modern allotetraploid genome of *G. hirsutum* and *G. barbadense* are allotetraploid generated by the hybridization of the two diploid progenitor species, which contributed to the A and D genomes. The number of *GhEC1* genes (14) and *GbEC1* genes (13) were generally consistent with the sum of six *GaEC1* genes and six *GrEC1* genes. This expansion in *EC1* genes indicates the important functions of *EC1* gene in cotton growth and development. These results provide significant insights into the evolution and functions of *EC1* genes in cotton. Gene duplication is a common phenomenon observed in virtually all plants [44]. Plants evolve to produce variations in gene family size, which allows evolution of gene functions but also introduces functional redundancy, resulting in increased genetic robustness. In this study, five pairs of whole-genome or segmental duplications among 14 *GhEC1* genes, five pairs among 13 *GbEC1* genes, six pairs between *G. hirsutum* and *G. arboreum*, two between *G. barbadense* and *G. raimondii* were identified. These results imply that whole-genome or segmental duplications may be one of the main driving forces of *EC1* family expansion during the evolution of cotton. In this study, most EC1 proteins contain a prolamin-like domain, though two domains were also present in some genes, suggesting that the function is highly conserved in the evolutionary process. EC1 proteins have six cysteines to form three pairs of disulfides bonds. Disulfide bonds are a typical feature of secreted proteins and subcellular localization could be a key factor in defining the function of genes. Bioinformatics analysis showed that subcellular location prediction of the *EC1* members was extracellular (Secreted) and none of the genes analyzed contained any predicted introns. Furthermore, combining the phylogeny of EC1 proteins with their peptide length, we found that EC1 is a highly conserved small cysteine-rich secreted protein that plays an important role in double fertilization.

Many nsLTPs (non-specific lipid-transfer proteins), DEFLs (defensin-like genes), and ECA1 gametogenesis-related family *CRPs* are preferentially expressed in reproductive tissues [45,46,47], indicating their role in enhancing different aspects of flowering plant reproduction. From the expression pattern of *GhEC1* genes in the different cotton tissues using the RNA-seq data and qRT-PCR (Figure 8), we found that *EC1* genes were highly expressed in ovules and anthers indicating their significant role in promoting the molecular mechanisms of plant flowering and reproduction. Protein interaction network analysis showed that regulation of double fertilization forming a zygote and endosperm pathway (NOG25365) was found around the interaction network. Gene interaction network analysis revealed that *EC1* genes may act as signals between the male gamete and the female gamete during fertilization. These results suggest that cotton *EC1* genes may participate in the process of gamete fertilization in plants. In plants, transcription factors bind to specific cis-elements to regulate the expression of genes and control diverse biological processes [48]. Many cis-elements related to stresses, plant growth, and development and phytohormone responses were extensively distributed in the promoter regions of *EC1* genes, implying the involvement of *EC1* genes in different biological processes. The light-related cis-elements and circadian elements in the promoter justified that the *EC1* genes might be involved in the circadian rhythm (Figure 4). Double fertilization in plants is a complex process in the fusion of the sperm and oocyte plasma membranes in which many genes play a role in the fusion of the egg cell with the sperm. Double fertilization is considered one of the most critical components of development because it ensures the growth, survival, and reproduction of flowering plants. In recent years, several small proteins involved in double fertilization have been identified, however, understanding of their role in plant is not fully elucidated. Many behaviors of male and female cells still have not found a reasonable mechanism for the whole double fertilization process. Identification and study of small peptide signal are the key processes to analyze the process of double fertilization. While this study provided biological insight into EC1 proteins function, future studies to ascertain the functional role of various important biological molecular mechanisms of this vital gene family in cotton are required.

## 5. Conclusions

The present study characterized the *EC1* gene family in cotton by a comprehensive sequence analysis, exon-intron structure, conserved motifs, phylogenetic relationships, gene collinearity, and expression profiles. The analysis lays a foundation for the study of *EC1* genes in cotton and other plants. However, the molecular mechanisms need to be verified and explored by more experiments in subsequent studies.

## Data Availability

The data presented in this study are available in the Supplementary Materials.

## Supplementary Materials

The following are available online at https://www.mdpi.com/article/10.3390/genes12122001/s1, Table S1: The details of *EC1* genes. Table S2: The primers used for qRT-PCR in this study. Table S3: Analysis of *EC1* cis-elements in cotton. Table S4: Synteny relationships among four cottons. Table S5: Expression values of *GhEC1* genes in tissues obtained from RNA-seq data. Table S6: Expression values of *GhEC1* genes in abiotic stresses obtained from RNA-seq data. Figure S1. Multiple sequence alignments of EC1 proteins from four cotton. (A) Prolamin-like-A domain region sequence alignment. (B) Prolamin-like-B domain region sequence alignment. Figure S2. Expression analysis of *GhEC1* genes in *G.* *hirsutum* TM-1 under abiotic stresses. The detail FPKM values are present in Table S5.
