# Supplementary figures and images for: The Evolution and Expression Profiles of EC1 Gene Family during Development in Cotton
Source: Genes (Basel). 2021 Dec 17;12(12):2001. doi: 10.3390/genes12122001 (PMC8702097; doi:10.3390/genes12122001)

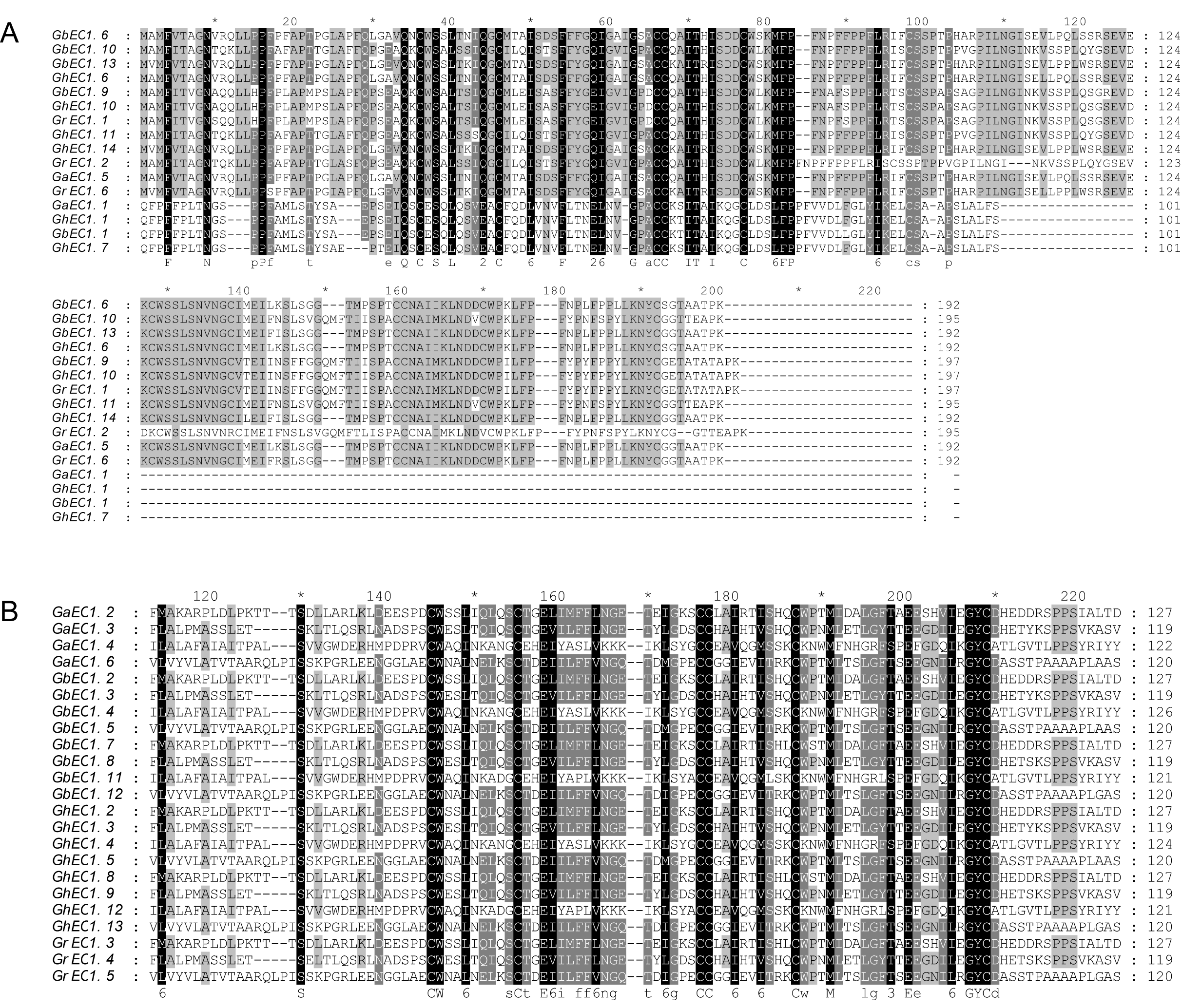

Supplement: Supplementary file 1 [file genes-12-02001-s001.zip › Supplementary data/Figure S1.tif]

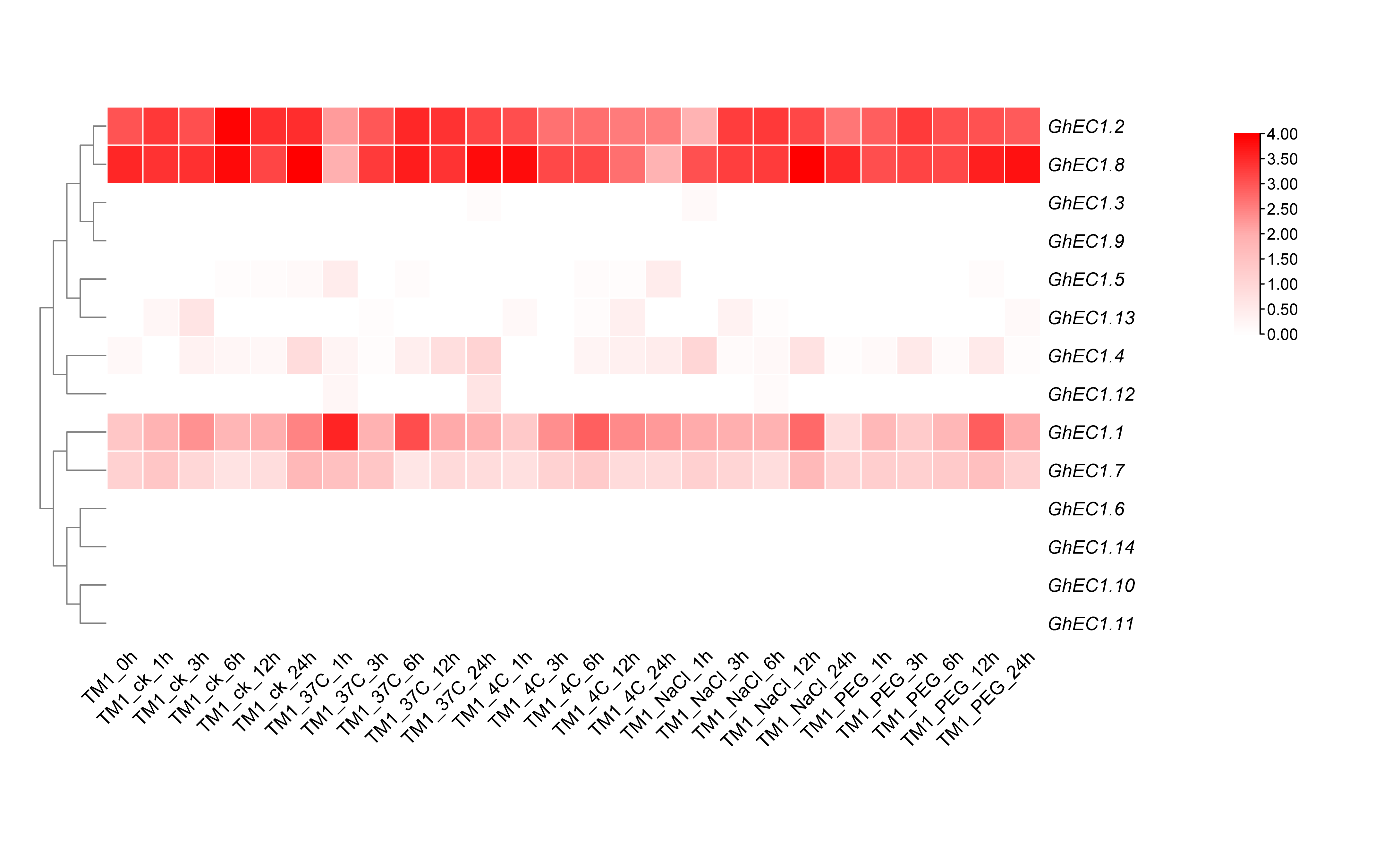

Supplement: Supplementary file 1 [file genes-12-02001-s001.zip › Supplementary data/Figure S2.tif]
